# A novel antimicrobial peptide M1‐8 targets the lysosomal pathway to inhibit autolysosome formation and promote apoptosis in liver cancer cells

**DOI:** 10.1111/jcmm.17644

**Published:** 2023-01-11

**Authors:** Jiali Zeng, Jian Wang, Jibin Wu, Rui Deng, Lun Zhang, Qingru Chen, Jie Wang, Xiaobao Jin, Shuiqing Gui, Yinghua Xu, Xuemei Lu

**Affiliations:** ^1^ Guangdong Provincial Key Laboratory of Pharmaceutical Bioactive Substances, School of Life Science and Biopharmaceutics Guangdong Pharmaceutical University Guangzhou China; ^2^ Intensive Care Unit, Shenzhen Second People's Hospital The First Affiliated Hospital of Shenzhen University Shenzhen China; ^3^ Key Laboratory of the Ministry of Health for Research on Quality and Standardization of Biotech Products National Institutes for Food and Drug Control Beijing China; ^4^ Central Laboratory of Shenzhen Center for Disease Control and Prevention Shenzhen China

**Keywords:** antimicrobial peptides, autophagy, liver cancer, lysosomes

## Abstract

Lysosomes, a central regulator of autophagy, play a critical role in tumour growth. Lysosomal protease cathepsin D can initiate apoptosis when released from lysosomes into the cytosol. In this study, we observed that *Musca domestica* cecropin (Mdc) 1–8 (M1‐8), a small anti‐tumour peptide derived from Mdc, inhibits hepatoma cell growth by blocking autophagy–lysosome fusion. This effect is likely achieved by targeting lysosomes to activate lysosomal protease D. Additionally, we examined whether lysosomal content and cathepsin D release were involved in M1‐8‐induced apoptosis. After exposure to M1‐8, human hepatoma HepG2 cells rapidly co‐localized with lysosomes, disrupted lysosomal integrity, caused leakage of lysosomal protease cathepsin D, caspase activation and mitochondrial membrane potential changes; and promoted cell apoptosis. Interestingly, in M1‐8‐treated HepG2 cells, autophagic protein content increased and the lysosome–autophagosome fusion was inhibited, suggesting that M1‐8 can cause apoptosis through autophagy and lysosomes. This result indicates that a small accumulation of autophagy and autolysosome inhibition in cells can cause cell death. Taken together, these data suggest a novel insight into the regulatory mechanisms of M1‐8 in autophagy and lysosomes, which may facilitate the development of M1‐8 as a potential cancer therapeutic agent.

## 1 INTRODUCTION

Liver cancer is a global health problem, representing the sixth most frequent malignancy in terms of incidence and the fourth most common cause of cancer‐related deaths worldwide. The major risk factors that contribute to hepatocellular carcinoma (HCC) are hepatitis B or C virus infection, alcohol abuse, intake of the microbial metabolite aflatoxin B1 and non‐alcoholic fatty liver disease (NAFLD). Conventional chemotherapeutic agents (e.g., doxorubicin, fluoropyrimidines, platinum derivatives and irinotecan) are inefficient in HCC treatment and do not improve patient survival.^1^, ^2^ Thus, development of novel strategies and anti‐tumour agents that can overcome cancer drug resistance and achieve enhanced efficacy is the major aim of current cancer research, including HCC.

Peptides are naturally produced by all organisms and exhibit a wide range of physiological, immunomodulatory and wound healing functions.^3^ Over the past few years, some studies have demonstrated that novel synthetic antimicrobial peptides can effectively inhibit growth and induce apoptosis in a wide variety of human malignant cells.^4^ Antimicrobial peptides (AMPs) are small molecules with effective tissue penetration, which can infiltrate the lipid bilayer membrane and cause breakdown of the transmembrane electrochemical gradient, resulting in the loss of energy for water and ion transport across the membrane, eventually promoting cell shrinkage and lysis, and thereby inhibiting the growth of cancer cells and bacteria.^4^, ^5^ The indirect mechanism involves the entry of peptides into the cell without disturbing the membrane, inhibiting protein synthesis or damaging the mitochondrial membrane, which results in the activation of the apoptotic pathway‐mediated by caspases.^6^ AMPs with dual antimicrobial and anticancer activities are promising therapeutic agents that can be used to combat HCC as a standalone treatment or as part of a synergistic treatment regimen.^7^ However, the use of peptides has limitations, including a short plasma half‐life, haemolysis and immunogenicity.^8^ Rational design of antimicrobial peptides can improve their stability and safety. Furthermore, the rational design of sequences for anticancer peptides involves fragmentation, which is a type of alteration of the original sequence into shorter sequences. For example, the 10 amino acids at the N‐terminus of cecropin can be used and repeated three times to create a CB1 peptide.^9^ It is also expected that shorter peptides can reach the membrane phospholipid bilayer more efficiently and reduce the polypeptides cytotoxicity.

*Musca domestica* cecropin (Mdc) is a linear molecule containing 40 amino acids, with three α‐helical structures appearing at residues 1–6, 9–21 and 27–39. Previous studies have demonstrated that Mdc has strong antibacterial and anticancer activity.^10^, ^11^, ^12^ The peptide's structure may restrict its internal properties and contribute substantially to production costs. To obtain a more economical, more effective, simpler, less toxic, more selective or more stable anticancer peptide, we simplified Mdc appropriately by retaining the sequence related to its anticancer activity.

M1‐8 (G‐W‐L‐K–K‐I‐G‐K) is derived from the N‐terminal 1–8 amino acids of Mdc. However, whether M1‐8 has an effective inhibitory effect on hepatoma cells is unclear. Furthermore, the precise inhibition mechanisms of M1‐8 remain unknown. Therefore, in this study, we aimed to investigate the effects and mechanisms of M1‐8 on liver cancer in vitro and in vivo.

## 2 MATERIALS AND METHODS

### 2.1 Peptide and materials

Mdc 1–8 (M1‐8) (purity: 97.15%; molecular weight: 4301.59 Da) was chemically synthesized using the conventional Fmoc solid‐phase synthetic method (Beijing SciLight Biotechnology Ltd.).^12^ Doxorubicin hydrochloride (Dox) at over 98% purity was purchased from MP Biomedicals LLC. The apoptosis inhibitor Z‐VAD was purchased from APExBIO.

### 2.2 Cell culture

HepG2, L02, Chang liver and NCM460 cells were purchased from the China Center for Type Culture Collection. The cells were cultured in Dulbecco's modified eagle medium (DMEM) or 1640 medium (Gibco) containing 10% heat‐inactivated fetal bovine serum (FBS) in a humidified CO_2_ incubator (5% CO_2_ in air) at 37°C.

### 2.3 MTT Assay

The viability of HepG2, L02 and NCM460 cells treated with the peptides was determined using the MTT assay. The cells were seeded in 96‐well plates at a density of 4 × 10^3^ cells/well. Then, 100 μl of peptide solutions of different concentrations were introduced into the wells, followed by further incubation for 24 h. Subsequently, 20 μl of MTT (5 mg/ml) was added to each well. After culturing for 4 h, the supernatant was discarded and the precipitated formazan was dissolved in 150 μl of dimethyl sulfoxide (DMSO). The absorbance at 490 nm (OD_490_) was measured using a microplate reader.

### 2.4 Cell migration assay

On the outsole of the six‐well plate, four lines were evenly spaced with a marker pen at 1‐cm intervals. HepG2 cells in the logarithmic growth phase were inoculated in a six‐well plate and cultured to 90% confluent state. The cells were cultured in a serum‐free medium for 24 h. The plates were scratched using a 10 μl pipette tip with a pattern of two lines perpendicular to each other. After 0, 24 and 48 h, the cells were observed and photographed under a microscope (Zeiss). The distance of scratches was then calculated, and the changes in migration distance were compared.^13^

(1) $$\text{Migration distance}=\text{blank distance}\;\left(0\;h\right)-\text{blank distance}\;\left(\text{dosing time}\right)$$

### 2.5 Cell invasion assay

Matrigel (Corning) was carefully added to each well at 37°C for 6 h. The cells were then diluted to 1 × 10^6^ cells/ml with serum‐free DMEM medium containing 0.1% BSA, and the transwell chamber was hatched with DMEM at 37°C for 30 min. The cell suspensions were held in the upper chamber, and 10% drug‐containing DMEM was added to the lower chamber. Subsequently, the cells were then incubated at 37°C under 5% CO_2_. After 48 h, the chamber was fixed with 100% methanol for 20 min, dyed with 0.1% crystalline methylene chloride for 15 min and rinsed with PBS. Five random areas were chosen, and the number of cells invading the membrane was counted.^14^

### 2.6 Cell apoptosis assay via flow cytometry

Annexin V (MultiSciences) was used to assess phosphatidylserine exposure, and propidium iodide (PI, MultiSciences) was used for cell viability assessment. The treated cells were harvested and stained using the Annexin V‐FITC/PI apoptosis kit or PI according to the manufacturer's instructions.^15^, ^16^ Cell apoptosis was analysed via flow cytometry (Bio‐Rad).

### 2.7 Inhibition of tumour growth in vivo

Male athymic Balb/c nude mice (body weight: 18–22 g, 4–6 weeks old) were obtained from the Guangdong Medical Laboratory Animal Center (GDMLAC). Animal use and the experimental protocols were approved by the Guidelines for the Care and Use of Experimental Animals, the Guangdong Pharmaceutical University (SYXK (Yue) 2012–0125) and the Guangdong Pharmaceutical University Animal Care and Use Committee, China. The mice were raised under specific pathogen‐free conditions. Every effort was made to minimize suffering. For tumour growth assays in subcutaneous xenograft nude mouse models, approximately 1 × 10^6^ HepG2 cells were collected, suspended in 0.1 ml PBS and injected subcutaneously into the forelimb flank of the Balb/c nude mice. Mice were randomly divided into three groups when the maximum diameter of the tumour reached approximately 5 mm. They were treated with 15 mg/kg of M1‐8 or Dox in the vicinity of the tumour once every 48 h for 2 weeks. An equal volume of saline was injected into the control mice. The length (L) and width (W) of the tumours were measured every 2 days. Tumour volume was calculated using the formula:

(2) $$V=\left(L\times W^{2}\right)/2$$

The mice were sacrificed on the second day after the last administration, and the tumours were removed for immunohistochemical analysis. The tumour inhibition rate was calculated as follows:

(3) $$\text{Inhibition rate}\;\left(\%\right)=\left[V_{\text{tumor}\left(\text{control}\right)}-V_{\text{tumor}\left(\text{treated}\right)}\right]/V_{\text{tumor}\left(\text{control}\right)}\times 100\%$$

### 2.8 Laser confocal detection of drug localization

The organelles were stained using MitoTracker® Red CMXRos and LysoTracker Red according to the manufacturer's instructions. Co‐localization with FITC‐labelled M1‐8 was observed using a laser scanning confocal microscope (NikonTi‐EA1).

### 2.9 Immunofluorescence staining and fluorescence microscopy

Cells were seeded on 6‐mm round glass coverslips and placed at the bottom of 24‐well plates. After M1‐8 treatment, cells on glass coverslips were fixed with cold methanol/acetone (1:1) for 5 min, followed by incubation with 10% BSA in PBS and 0.5% Triton X‐100 for 20 min. After washing, the cells were incubated with primary antibodies and immunostained with fluorescent‐labelled secondary antibodies. The DNA was counterstained with DAPI for 15 min at 25°C. Fluorescence images were captured using a fluorescence microscope (Zeiss).

The intralysosomal pH was estimated using LysoTracker as per the manufacturer's instructions.^17^ The fluorescence intensity was observed under a fluorescence microscope (Leica, Switzerland) and photographed.

For autophagic flux analysis, HepG2 cells were seeded and grown overnight on a 20‐mm glass‐bottom cell‐culture dish. The formation of autolysosomes in cells treated with M1‐8, Dox and chloroquine (CQ) was detected using a Beyotime Autophagy Tandem Sensor Ad‐mCherry‐GFP‐LC3B Kit (Beyotime, China) according to the manufacturer's protocol.

The cells were fixed with 4% paraformaldehyde for 20 min. Then, the cells were stained with Hoechst 33258 (2 μg/ml) in the dark for 15 min at 25°C. All fluorescent images were captured using a fluorescence microscope and quantified using Image J software.

### 2.10 Transmission electron microscope assay

After treatment with M1‐8 (400 μg/ml) for 24 h, the cells were pelleted via centrifugation and diced into blocks <1 mm^3^, which were placed in fresh fixative (1% OsO_4_) for 2 h at 4°C. Subsequently, the blocks were dehydrated using an ascending series of alcohol solutions. The samples were stained with 2% uranyl acetate for 30 min. The samples were then embedded in an epoxy resin overnight at 60°C. Following polymerization, the blocks were cut into 0.5‐mm thick slices, stained with 1% toluidine blue and observed using a JEM1400 (Jeol) transmission electron microscope.^18^

### 2.11 Quantitative reverse transcription PCR (qPCR)

After administration of M1‐8 or Ah for 48 h, the cells in all groups were collected to determine the mRNA level. All primers were purchased from the Invitrogen Trading Company. The primer sequences are shown in Table 1. The RNAiso Plus extraction method was used to extract total RNA from the cells (TaKaRa). Reverse transcription was performed using a PrimeScript RT Reagent Kit (TaKaRa).^16^, ^19^ qPCR reaction was performed using SuperReal PreMix Plus (TIANGEN) in a Bio‐Rad RT‐PCR thermocycler (Bio‐Rad). The genes in the cells were calculated based on the Ct values. The results are presented as changes in M1‐8‐treated vs. control.^19^

**TABLE 1:** Primers used in this study

Gene		Primer sequence(5′–3′)
GAPDH	Forward Primer	CTCTGCTCCTCCTGTTCGAC
	Reverse Primer	ACGACCAAATCCGTTGACTC
TSC1	Forward primer	TACTCCCATAGACCTGCCCT
	Reverse primer	ATGGGCTGTCTTTGGCAATG
Beclin	Forward primer	AATCTCGAGAAGGTCCAGGC
	Reverse primer	TTGTGCCAAACTGTCCACTG

### 2.12 Isolation of lysosomes

Lysosomes were isolated using the Lysosome Enrichment kit according to the manufacturer's instructions (Thermo‐Fisher Scientific, USA). Cathepsin D proteins within lysosomes were detected by Western blot.

### 2.13 Western blot

HepG2 cells and lysosomes were lysed using RIPA peptide lysis buffer (Beyotime) containing 1% protease inhibitor (Beyotime). The protein content of different fractions was detected via the BCA method. Equivalent amounts of protein (20 μg) were separated on 10% SDS‐PAGE gels, transferred to nitrocellulose membranes and blocked with 1% BSA in TBST for 1 h at 25°C. The membranes were incubated with BAX, Bcl‐2, P53, P62, Cleaved PARP, Cleaved Caspase 3, LC3 and Cathepsin D (Abcam) antibodies overnight at 4°C. After washing, the membranes were incubated with an HRP‐conjugated secondary antibody (1:1000; Beyotime) for 1 h. Images were taken with Tanon‐5200 (Shanghai Tanon Technology Co. Ltd.) for protein level quantification; appropriate film exposures were scanned. The density of bands was determined with ImageJ and normalized to band intensity for GAPDH or β‐Actin.

### 2.14 Histology and immunohistochemistry assays

The excised tumours were fixed in 4% paraformaldehyde, paraffin‐embedded, sectioned at 5 μm thickness and prepared for H&E staining or tumour proliferation antigen P53 and Caspase‐3 assays. Immunohistochemical staining was performed according to the manufacturer's instructions. The heart, liver, spleen, lung, kidney and tumour tissues of the mice bearing orthotopic xenograft tumours were harvested at the end of the experiment. The samples were excised, fixed with 10% neutral phosphate‐buffered formalin and embedded in paraffin. Continuous sections (5 μm thick) were obtained and stained with H&E for histomorphometric analysis.

### 2.15 Statistical analysis

Data are presented as mean ± standard errors. Statistical significance was analysed using the unpaired two‐tailed Student's *t*‐test for at least three independent experiments using GraphPad Prism (GraphPad Software). The *p*‐value <0.05 was considered statistically significant. *p < 0.05; **p < 0.01; ***p < 0.001.

## 3 RESULTS

### 3.1 The effect of M1‐8 on proliferation, migration and invasion of cells

**FIGURE 1:**
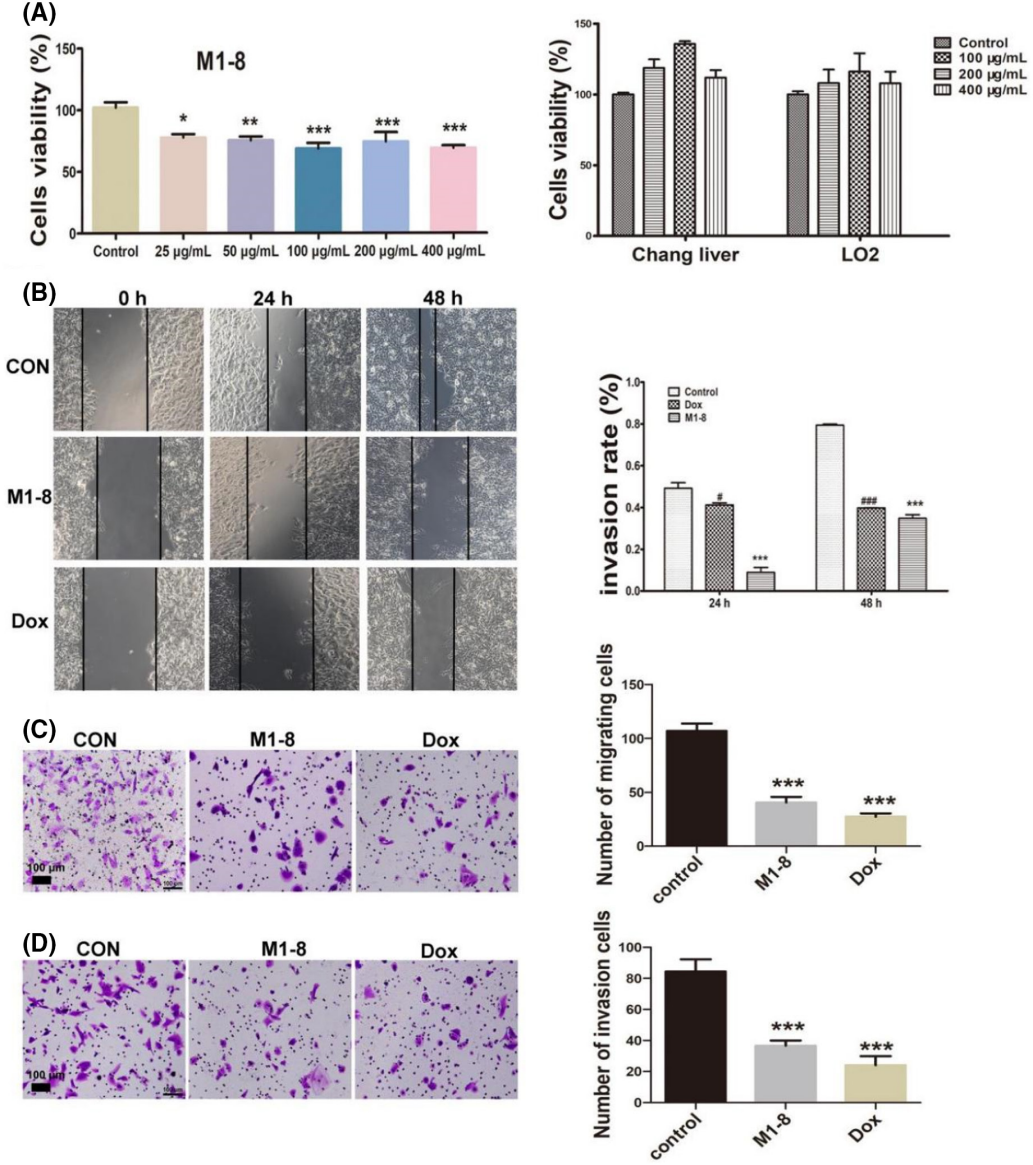
The effect of M1‐8 on proliferation, migration and invasion of cells. (A) Cytotoxicity of M1‐8 on HepG2, Chang liver and L02 cells. (B) Scratch assay was performed to detect the effect of M1‐8 on HepG2 cell migration. (C) The migration of HepG2 cells was investigated via transwell assay. (D) The invasion of HepG2 cells was investigated via transwell assay. *p < 0.05, **p < 0.01, ***p < 0.001 as compared with the negative control group. ^#^ *p* < 0.05, ^###^ *p* < 0.001 as Dox control group compared with the negative control group.

We synthesized a novel derivative of Mdc, that is, M1‐8. Thereafter, we tested the inhibition of cell growth in three human cell lines (HepG2, L02 and Chang liver) to determine whether M1‐8 can be used as a new therapeutic compound. The activity of the peptides on cancer cells HpeG2 and normal cells, including L02 and Chang liver cells, was examined using the MTT assay. The results are summarized in Figure 1A. M1‐8 at an 400 μg/ml concentration showed no obvious inhibition of proliferation in these two normal cell lines. Conversely, M1‐8 at 25 μg/ml concentration suppressed the proliferation of HepG2 cells. The influence of M1‐8 on cell migration ability is shown in Figure 1B. The results indicated that the scratches of M1‐8 treated groups healed more slowly than those of the control group, and the migration distance was reduced in a dose‐dependent manner (*p* < 0.05). Treatment with M1‐8 could reduce the migration ability of HepG2 cells. The results of the transwell migration assay showed that M1‐8 could decrease the migration ability of HepG2 cells (Figure 1C). The effect of M1‐8 on cell invasion was detected via a transwell assay (Figure 1D). The results revealed that M1‐8 inhibited the invasion of HepG2 cells in the Matrigel‐coated transwell membranes. These results indicated that M1‐8 could inhibit the proliferation and metastatic progression of HepG2 cells.

### 3.2 M1‐8 affected cell autophagy and apoptosis

**FIGURE 2:**
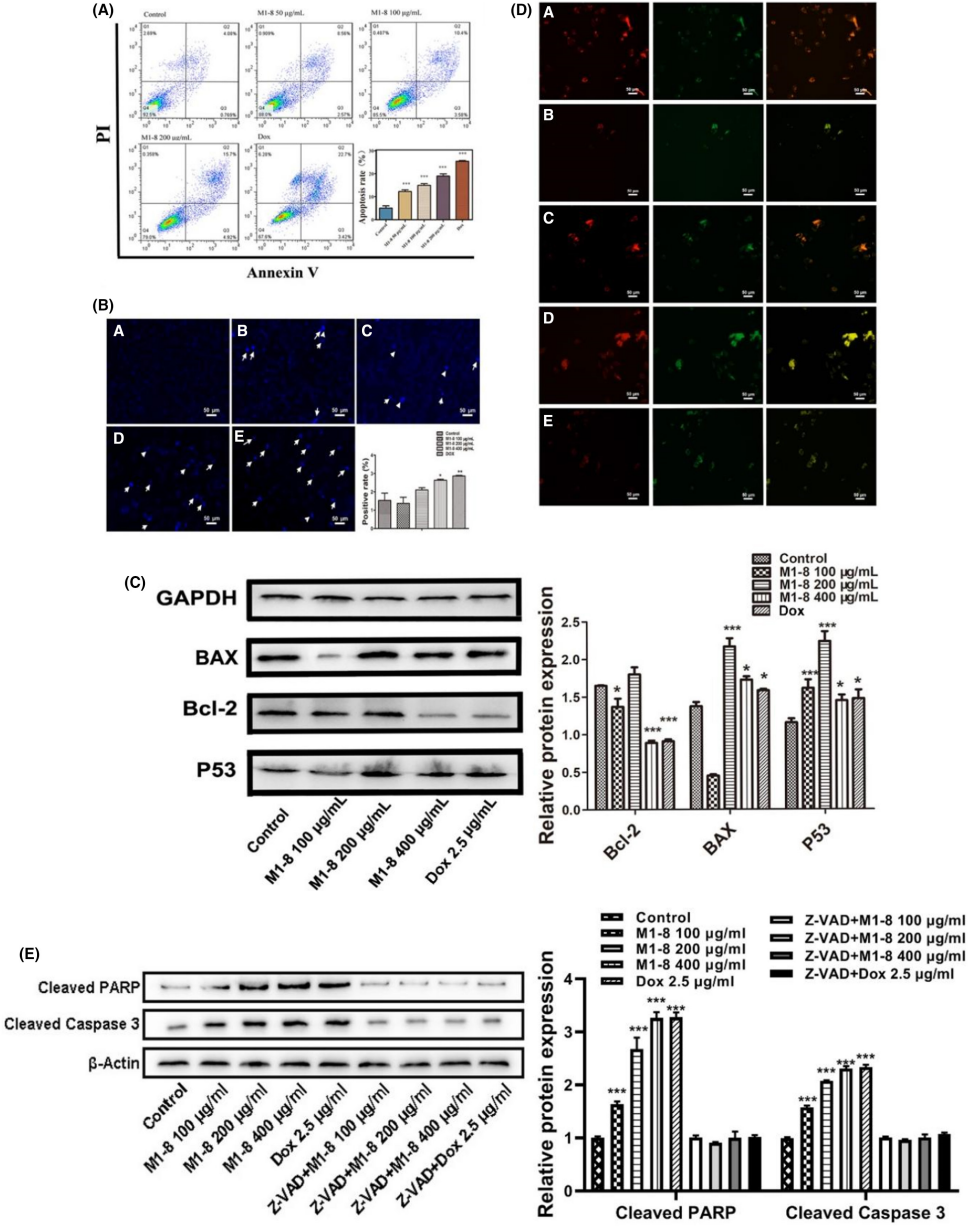
M1‐8 promotes apoptosis in HepG2 cells. (A) M1‐8 on HepG2 cell apoptosis. (B) Morphological observation of HepG2 cells treated with M1‐8 (A) Control, (B) M1‐8100 μg/ml, (C) M1‐8200 μg/ml, (D) M1‐8400 μg/ml, (E) Dox. (C) The expression of apoptosis‐related proteins in HepG2 cells treated with M1‐8 was determined by Western blot. (D) The JC‐1 fluorescence staining of HepG2 cells treated with M1‐8. (A) Control, (B) Dox 2.5 μg/ml, (C) M1‐8100 μg/ml, (D) M1‐8200 μg/ml, (E) M1‐8400 μg/ml. (E) The expression of Cleaved PARP and Cleaved Caspase‐3 in HepG2 cells treated with M1‐8 and Z‐VAD + M1‐8 were determined by Western blot. *p < 0.05, **p < 0.01, ***p < 0.001 as compared with the negative control group.

HepG2 cancer cells treated with specified doses of M1‐8 were stained with Annexin V‐FITC/PI and then analysed via flow cytometry to investigate whether M1‐8 induced cell death in cancer cells through apoptosis. Annexin V^+^/PI^−^ is a hallmark of early apoptosis. Annexin V^+^/PI^+^ cells represent the end‐stage of apoptosis. As shown in Figure 2A, M1‐8 treatment caused a significant increase in the fraction of apoptotic cells in a dose‐dependent manner, particularly the percentage of apoptotic cells, which increased from approximately 4% to 25% in HepG2 cells. Hoechst 33258 staining was used to detect nuclear morphology (Figure 2B). Cells treated with M1‐8 showed increased cell apoptotic nuclei characterized by nuclear fragmentation and chromatin condensation.

A decrease in the mitochondrial membrane potential is an early sign of apoptosis. In normal cells, JC‐1 produces red fluorescence in the mitochondrial matrix and green fluorescence in the cytoplasm after staining with JC‐1 working fluid. A decrease in red fluorescence and an increase in green fluorescence were observed in the M1‐8 group. This phenomenon indicated a decrease in mitochondrial membrane potential and the occurrence of early apoptosis in the M1‐8 treated group (Figure 2D). Furthermore, we analysed the effect of M1‐8 on the expression of apoptotic proteins, including BAX, Bcl‐2, P53, Cleaved PARP and Cleaved Caspase‐3. Western blot results showed that M1‐8 reduced the expression of the apoptosis inhibitory protein Bcl‐2 and increased the expression of the pro‐apoptotic proteins BAX and P53 (Figure 2C). Besides, M1‐8 increased the expression of the apoptosis protein Cleaved PARP and Cleaved Caspase‐3 in HepG2 cancer cells in a dose‐dependent manner (Figure 2E). When HepG2 cells treated with Z‐VAD + M1‐8, the results showed that M1‐8 did not change the expression of Cleaved PARP and Cleaved Caspase‐3 (Figure 2E). These results indicated that M1‐8 could promote the apoptosis of HepG2 cells by activating P53/Caspase‐3 signalling pathway.

### 3.3 M1‐8 accumulated in the lysosomes

**FIGURE 3:**
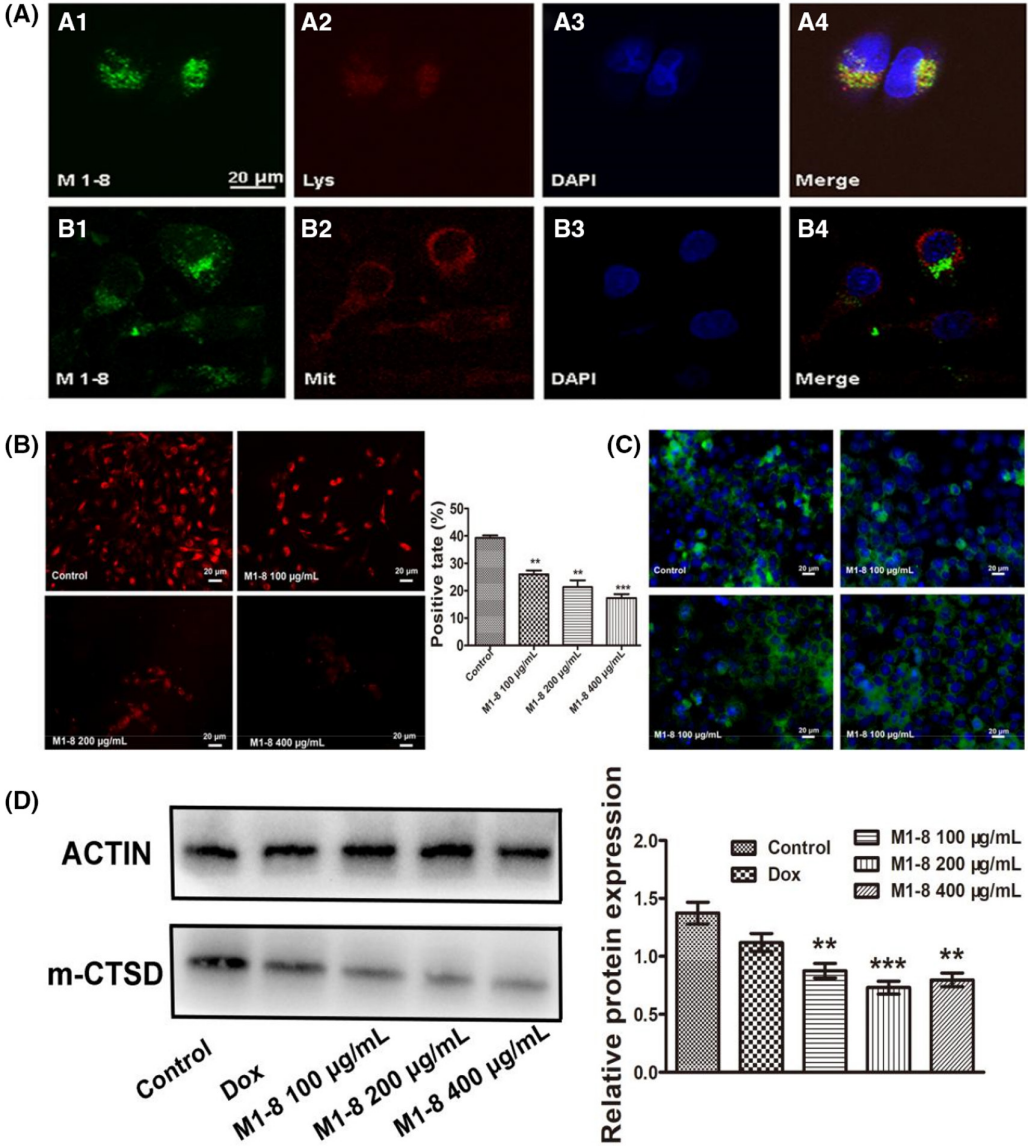
M1‐8 accumulated in the lysosomes. (A) Laser confocal detection of M1‐8 localization in HepG2 cells. (A) The lysosome group, (B) The mitochondrion group. (B) The lysosomes of HepG2 cells treated with M1‐8 were analysed by fluorescence microscopy. (C) The cathepsin D of HepG2 cells treated with M1‐8 were analysed by fluorescence microscopy. (D) The expression of m‐CTSD protein in HepG2 cytolysin was determined by Western blot. *p < 0.05, **p < 0.01, ***p < 0.001 as M1‐8 group compared with the negative control group.

Co‐localization of M1‐8 with organelles was examined via laser confocal microscopy. LysoTracker Red, a fluorescent weak base that accumulates in acidic organelles, can successfully stain lysosomes in living cells.^20^ As a green fluorescent substance, FITC groups chemically linked to M1‐8 can cause M1‐8 to fluoresce green. Under a laser confocal microscope, the intracellular localization of LysoTracker Red and FITC‐labelled M1‐8 was visualized as yellow (Figure 3A). Mito Tracker® Red CMXRos can specifically label the bioactive mitochondria in cells, and Figure 3A shows that the active mitochondria inside the cells emit red fluorescence and FITC‐labelled M1‐8 emit green fluorescence inside the cells, both of which are clearly coloured without evident overlapping sites via laser confocal microscopy. According to the result that M1‐8 colocalized with mitochondria and lysosomes, we found that M1‐8 was mainly enriched in lysosomes after entering the cells. Lysosomal staining by LysoTracker Red showed that the fluorescence intensity of lysosomes decreased with the increase in M1‐8 concentration (Figure 3B). Lysosomal rupture after 2 h of M1‐8 treatment was indicated by the increased numbers of “pale cells,” that is, cells with reduced numbers of lysosomes. Immunofluorescence detection of lysosomal cathepsin D showed that it had a different distribution pattern within the negative control and M1‐8‐treated cells (Figure 3C). The negative control cells showed distinct granular staining, whereas M1‐8 treated cells showed a diffuse pattern. This difference may have resulted from the enzyme release from lysosomes into the cytoplasm. The major role of the mature cathepsin D (m‐CTSD) is the digestion of internalized waste cell proteins and peptides in the lysosome, which maintains cell health. In the cytosol, m‐CTSD has a pro‐apoptotic function. To confirm the involvement of cathepsin‐D, we also detected the expression of m‐CTSD within lysosomes of HepG2 cells after M1‐8 treatment. The lysosomal extract of M1‐8 treated HepG2 cells showed reduced m‐CTSD level compared to the control group (Figure 3D). The above results indicated that M1‐8 can disrupt the integrity of lysosomes in HepG2 cells, leading to the release of m‐CTSD into the cytosol and inducing apoptosis.

### 3.4 Effect of antimicrobial peptides M1‐8 on autophagy in HepG2 cells

**FIGURE 4:**
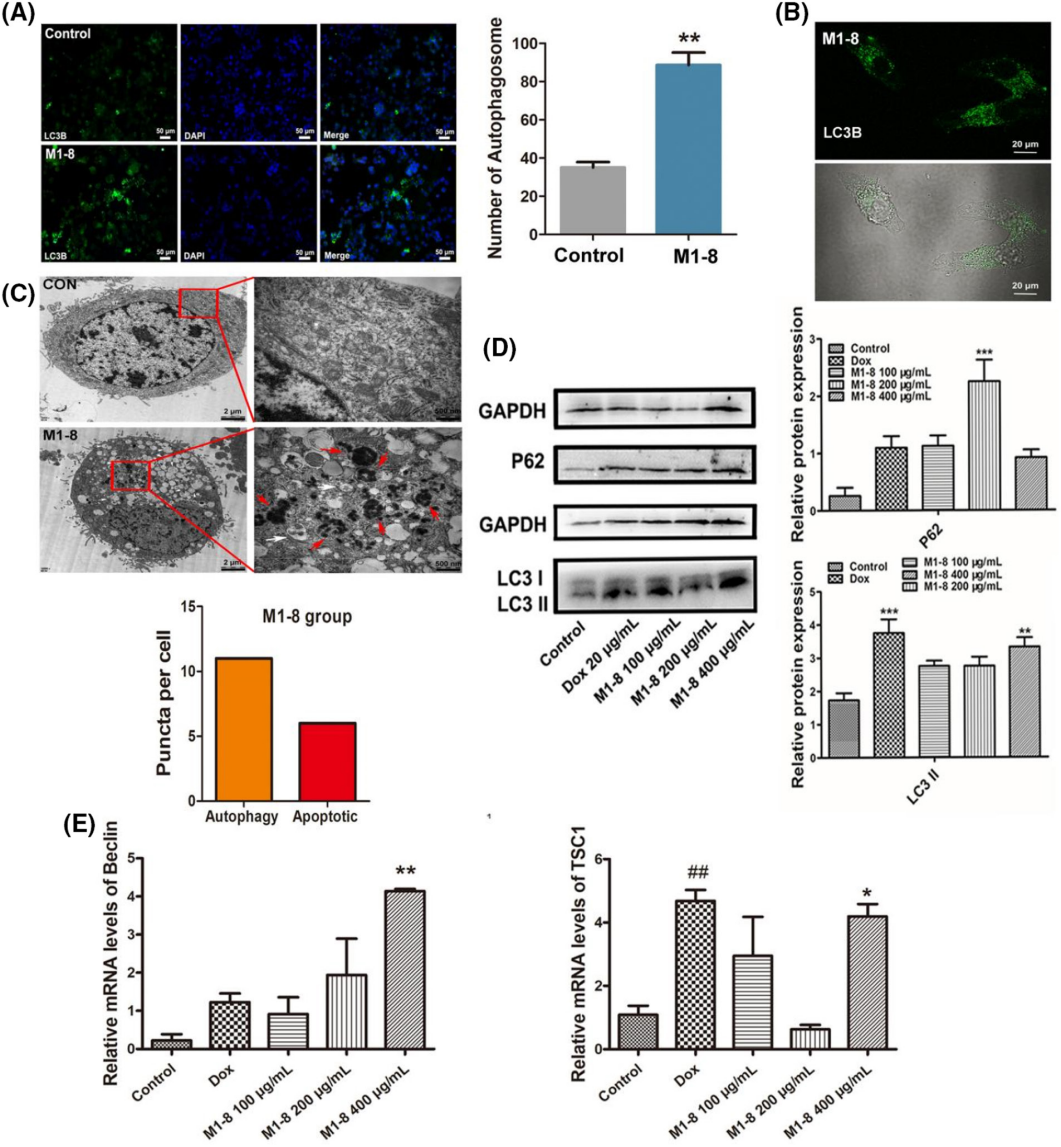
Effect of M1‐8 on autophagy in HepG2 cells. (A) Autophagy was induced by M‐8 in HepG2 cells via fluorescence microscopy. (B) M1‐8 induced autophagy in HepG2 cells was observed by laser confocal microscopy. (C) The transmission electron microscopy of HepG2 cells treated with M1‐8. CON: Control group, M1‐8: M1‐8 group (white – autophagic features, red–apoptotic features). (D) The expression of autophagy‐related proteins in HepG2 cells treated with M1‐8. (E) Effects of M1‐8 on the mRNA expression of autophagy‐related genes Beclin and TSC1 in HepG2 cells. *p < 0.05, **p < 0.01, ***p < 0.001 as M1‐8 group compared with the negative control group. ^##^ *p* < 0.01 as positive control group compared with the negative control group.

Under fluorescence microscopy and laser confocal microscopy, the green fluorescence‐labelled LC3 protein in the M1‐8 group showed an aggregate spot‐like distribution. In contrast, the green fluorescence in the negative control group showed a diffuse distribution. The green fluorescence labelled LC3 protein in the M1‐8 group showed an aggregate spot‐like distribution, whereas the green fluorescence in the negative control group showed a diffuse distribution (Figure 4A). The fluorescence intensity in the drug administration group was considerably stronger than that in the negative control group, indicating that M1‐8 could increase LC3 II protein expression. Laser confocal microscopy revealed the green puncta formation by LC3 of the M1‐8 group (Figure 4B).

The microstructure of HepG2 cells was observed using a transmission electron microscope (TEM) (Figure 4C). The cell membrane of the negative control group was intact, the nucleus was regular and intact, the structure of each organelle was not abnormal, the cytoplasmic distribution was even, and no obvious vacuoles appeared in the cell. In the M1‐8 group, the microvilli on the cell surface appeared detached, the cell membrane appeared shrunken, the number of autophagosomes in the cell increased, and the nucleus appeared fragmented on one side.

Next, we examined the effects of M1‐8 on autophagy‐related proteins in HepG2 cells. As shown in Figure 4D, M1‐8 could cause significant changes in the contents of related proteins, such as autophagy in cells. P62 protein increased gradually with the increase in the concentration of the administered M1‐8, whereas M1‐8 markedly promoted LC3 II content and downregulated LC3 I protein expression. At the same time, Beclin and TSC1 gene expression in the M1‐8 group was upregulated with the increase in the concentrations of the administered drugs compared to the negative control group, indicating that the M1‐8 mechanism of action is related to autophagy (Figure 4E).

### 3.5 Effects of M1‐8 on autophagic flux in HepG2 cells

**FIGURE 5:**
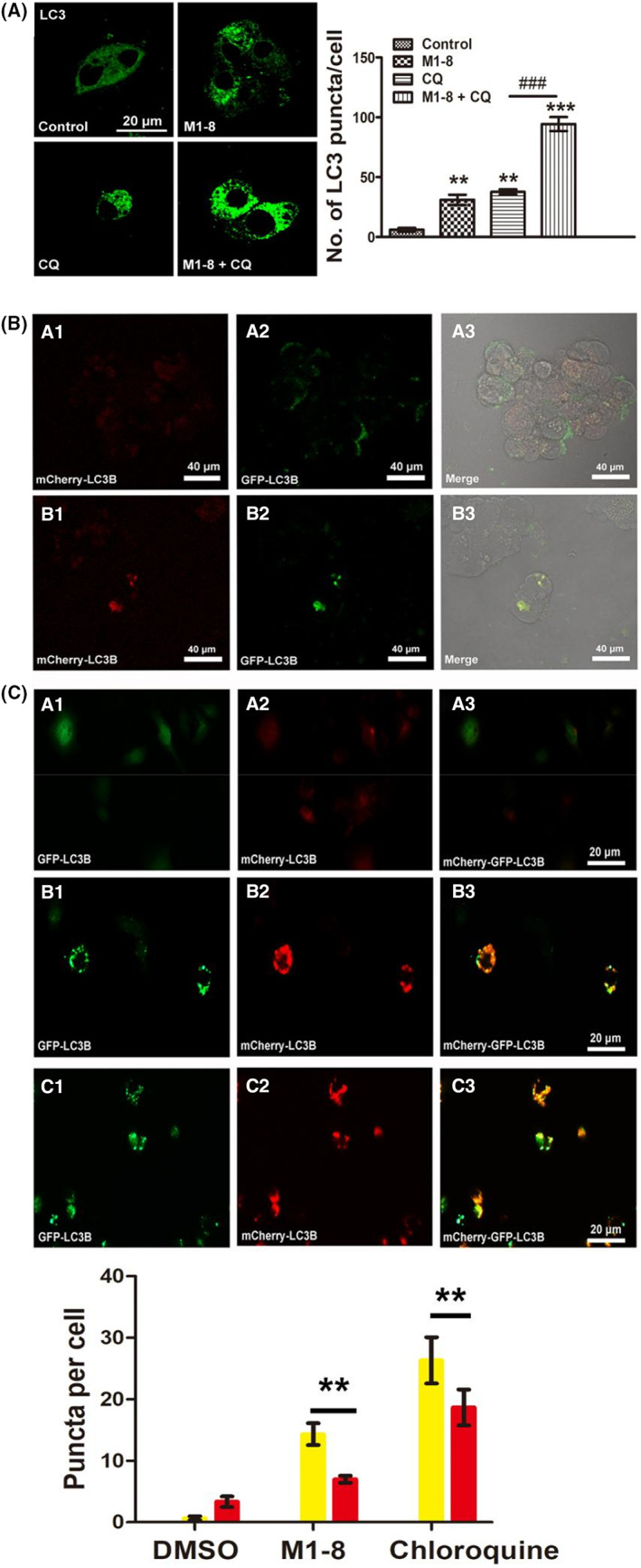
Effects of M1‐8 on autophagic flux in HepG2 cells. (A) The accumulation of LC3 puncta was examined by immunofluorescent analysis of cells treated with CQ in the presence or absence of M1‐8 for 24 h. The number of LC3 puncta was quantitated. (B) Detection of autophagic flux in HepG2 cells by laser confocal microscope. (A) DMSO group, (B) M1‐8 group. (C) Detection of autophagic flux in HepG2 cells by fluorescence microscopy. (A) DMSO group, (B) M1‐8 group, (C) Chloroquine group. **p < 0.01, ***p < 0.001 as M1‐8 groups, CQ group and M1‐8 + CQ group compared with the negative control group. ^###^ *p* < 0.001 as M1‐8 + CQ group compared with the CQ group.

As shown in Figure 4D, we observed increased levels of p62 as well as increased levels of LC3 II in M1‐8 treated cells, implying that M1‐8 could induce an increase in autophagosomes but complete autophagic flux might be blocked. In addition, combinatorial treatment of M1‐8 with autolysosome inhibitors CQ resulted in enhanced LC3 II turnover and accumulation of endogenous LC3 puncta (Figure 5A). MCherry and GFP can bind to the LC3 protein, with red fluorescence spreading after mCherry staining and green fluorescence spreading after GFP staining. GFP in both is easily quenched in low pH environments when the lysosome and autophagosome are combined, and the overall environment of the autophagosome becomes acidic so that only mCherry is present. Red and green fluorescence were presented as yellow fluorescence after superimposition, and the patency of autophagic flux within cells was observed according to the ratio of red and green puncta after superimposition. The more obvious red puncta after colour overlay suggested patency of autophagic flux, and the more yellow puncta indicated that the downstream channels of autophagic flux were inhibited. Fluorescence microscopy revealed more yellow autophagic puncta than red puncta in the M1‐8 group (Figure 5B,C). The experimental results illustrated that M1‐8 could promote autophagosome accumulation, but the downstream channels of autophagic flux were inhibited. Hence, the autophagosomes and lysosomes could not fuse normally.

### 3.6 In vivo anti‐tumour efficacy

**FIGURE 6:**
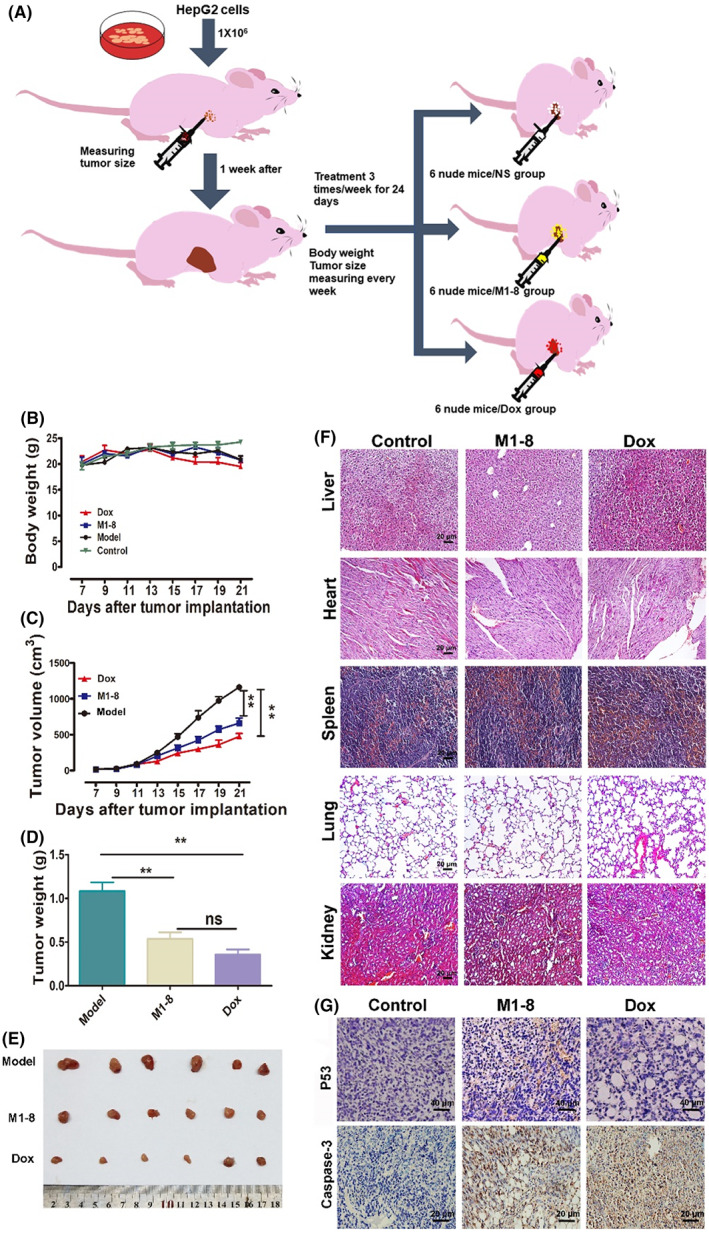
In vivo anti‐tumour efficacy. (A) The experimental design and arrangement. (B) The changes of body weight index in mice. (C) Tumour growth volume of tumour tissues in nude mice. (D) Tumour growth weight of tumour tissues in nude mice. (E) Subcutaneous transplantation of tumours in nude mice. (F) H&E staining of the liver, heart, spleen, lung and kidney. (G) Detection of P53 and Casepase‐3 by IHC. **p < 0.01, as M1‐8 and Dox group compared with the negative control group.

The results of the experiments are shown in Figure 6A. Although no significant difference was observed between the body weight of nude mice in each administration group and the normal control group, the body weight of nude mice in the normal group increased slightly with time. The body weight change curves of the M1‐8 group and the model group were similar, and the curve of the Dox group was lower than that of the other groups (Figure 6B). Tumour size was measured daily using vernier callipers, and the tumour growth curve was plotted. The tumour growth rate of the model group was the fastest, and the tumour volume of the M1‐8 group was smaller than that of the model group (Figure 6C). At the end of drug administration, the tumours of each group were peeled, photographed and weighed to show that the model group had the heaviest tumours, and the M1‐8 and Dox groups had significant differences in tumour weight compared to the model group (Figure 6D,E).

Morphological features of the liver, heart, spleen, lung and kidney were visualized in each group using haematoxylin and eosin (H&E) staining (Figure 6F). Histological examination revealed an increasing number of disintegrated cells in the presence of M1‐8 or Dox. The p53 and caspase‐3 antigen content in the tumour tissues of the M1‐8 and dox groups was substantially higher than that in the model group (Figure 6G). However, M1‐8 did not considerably damage the major tissues and organs of the nude mice.

## 4 DISCUSSION

Natural antimicrobial peptides have long sequences, complex structures and a high cost of artificial synthesis. With the advancement in research, natural antimicrobial peptides have gradually become popular research targets. Researchers have explored and improved the use of peptide derivatives for anti‐tumour research. M1‐8 is a peptide derived from the housefly antimicrobial peptide Mdc that was screened and found to have good in vitro anticancer activity. We used M1‐8 to cause cytotoxic effects and cell death in HepG2 cancer cells. In contrast, M1‐8 did not show significant cytotoxicity toward normal hepatocytes. We further investigated the effects of M1‐8 in vivo. We constructed a nude mouse HepG2 xenograft model for the in vivo studies. We observed that M1‐8 substantially inhibited tumour growth. Combining the results of in vitro and in vivo studies, we identified that the derived peptide M1‐8 inhibits the proliferation of HepG2 cells and suppresses tumour growth in vivo.

This is the first study to provide clear evidence that M1‐8 induces the accumulation of autophagosomes by inhibiting autophagosome–lysosome fusion. Autophagy is a basic catabolic metabolism that involves the degradation of unnecessary or dysfunctional cellular components through the action of lysosomes.^21^ Autophagy plays a major role in the degradation of damaged organelles and old proteins and the maintenance of cellular homeostasis.^22^, ^23^, ^24^ Lysosomes are acidic organelles that contain hydrolytic enzymes and are involved in several cellular processes, including post‐translational protein maturation, receptor degradation and extracellular release of active enzymes.^25^, ^26^, ^27^ Autophagy can be either general (nonselective) or selective. General autophagy packages portions of the cytoplasm into autophagosomes and delivers them to lysosomes for degradation.^28^ Several studies have demonstrated that autophagy can be inhibited pharmacologically by targeting different stages of the autophagic process.^21^ Our study shows that M1‐8 can inhibit autophagic lysosomal fusion based on the following findings: M1‐8 accumulates in lysosomes after cellular uptake and leads to lysosomal rupture, resulting in M1‐8 cytotoxicity towards HepG2; M1‐8 can inhibit the formation of autolysates, and it substantially accumulates autophagic substrates. Furthermore, cathepsins are always involved in cell death associated with lysosomes.^29^, ^30^ M1‐8 induced cell death may also be associated with lysosomal rupture and the leakage of histone proteases. M1‐8 primarily targets lysosomes and induces lysosomal leakage or rupture. M1‐8 is a small peptide with amphipathic properties that can be ingested by HepG2 cells and accumulates in large amounts in lysosomes. M1‐8 may directly target lysosomes and induce lysosomal leakage or partial rupture, leading to the release of cathepsin D from the lysosomes to the cytosol. The released cathepsin D may convert certain unknown substances to bioactive molecules, such as free radicals, which may attack other cellular organelles (e.g., mitochondria and nucleus). Additionally, cathepsin D may directly attack organelles or activate caspases. The mitochondria may undergo apoptotic alterations due to the attack of cathepsin D and/or free radicals. Therefore, apoptotic factors such as cytochrome C and caspases are released or activated to execute apoptosis.^31^ To prove this hypothesis, follow‐up experimental research is required. Nevertheless, our findings do not exclude the possibility that multiple pathways may be involved in autophagy.

## 5 CONCLUSIONS

In summary, the advantages of using the derivative M1‐8 from Mdc include, but are not limited to, the following: First, M1‐8 improved anti‐tumour activity in vitro and in vivo. The MTT assay revealed that M1‐8 exhibited excellent antiproliferative activity. Additionally, in mice with pre‐established tumours, M1‐8 substantially delayed the growth of HepG2 xenografts and was considerably effective in controlling tumour growth. Second, M1‐8 exhibited a different anticancer mechanism. Previously, Mdc has been shown to inhibit the growth of liver cancer cells. However, M1‐8 primarily targeted lysosomes and induced lysosomal leakage or rupture by inhibiting the fusion of autophagosomes and lysosomes.

This is the first study to demonstrate that M1‐8 potently inhibited autophagosome–lysosome fusion, leading to autophagosome accumulation. Recent progress in cancer research using cancer cell lysosomes has encouraged researchers to devote more effort into elucidating lysosomal death pathways and putative anticancer peptides, such as M1‐8. Further studies should be conducted to determine the molecular basis of M1‐8 activity in cancer cells.

## AUTHOR CONTRIBUTIONS

Jiali Zeng involved in conceptualization, data curation, formal analysis and writing the original draft. Jian Wang involved in review and editing, supervision and formal analysis. Jibin Wu involved in methodology and data curation. Rui Deng involved in data curation, validation and methodology. Lun Zhang and Qingru Chen involved in methodology and data curation. Jie Wang involved in funding acquisition and resources. Xiaobao Jin involved in resources, and project administration. Shuiqing Gui involved in conceptualization, funding acquisition, project administration and resources. Yinghua Xu involved in funding acquisition, project administration and resources. Xuemei Lu involved in conceptualization, funding acquisition, project administration, resources and methodology.

## FUNDING INFORMATION

This work was financially supported by the National Key R&D Program of China (No. 2018YFC1603900); National Natural Science Foundation of China (No. 32070509 and 31,501,894); Guangdong Basic and Applied Basic Research Foundation (No. 2021A1515220119); Guangdong Natural Science Foundation (No. 2020A1515011097); Shenzhen Science and Technology Program (No. JSGG20220606141800001); Shenzhen Fundamental Research Program (No. JCYJ20220530150412027).

## CONFLICT OF INTEREST

The authors declare no conflict of interest.

## Data Availability

Our data has not been shared previously and is not under consideration for publication elsewhere, in whole or in part.
